# Active learning in diabetes education for health professions students: a narrative synthesis of effectiveness and implementation considerations

**DOI:** 10.1080/10872981.2026.2656830

**Published:** 2026-04-22

**Authors:** Hui Zhou, Nie Tang, Limei Liu, Ying Zhu

**Affiliations:** a Department of Endocrinology, Sichuan Provincial People’s Hospital, School of Medicine, University of Electronic Science and Technology of China, Chengdu, Sichuan, People's Republic of China

**Keywords:** Diabetes, students, nursing, learning, narrative synthesis

## Abstract

**Background:**

Diabetes is a growing global health burden, requiring future health professionals to develop strong knowledge, clinical reasoning, and patient education skills. Active learning approaches, such as simulation, flipped classrooms, and gamification, are increasingly used to strengthen diabetes education, yet evidence remains fragmented.

**Objective:**

To synthesize recent evidence on the effectiveness and implementation of active learning strategies in diabetes education for pre-licensure health professions students.

**Methods:**

We conducted a structured narrative review of peer-reviewed studies published between 2010 and 2025. Eligible studies included randomized controlled trials, quasi-experimental, and pre-post designs evaluating active learning interventions in pre-licensure medical, nursing, or pharmacy programs. Outcomes included knowledge, skills, confidence, engagement, and satisfaction. Study quality was appraised using MERSQI.

**Results:**

Twenty studies met inclusion criteria (10 RCTs, 3 quasi-experimental, 7 pre-post). Simulation and gamification demonstrated the most consistent improvements in knowledge, skills, and learner engagement. Flipped classroom results were mixed, with effectiveness dependent on learner preparation and integration with experiential components. Technology-enhanced experiential approaches showed promise but faced scalability and resource challenges. Implementation factors including faculty time, infrastructure, and equity considerations substantially influenced outcomes.

**Conclusions:**

Active learning strategies enhance diabetes education for health professions students, with simulation and gamification showing the strongest evidence of effectiveness. However, sustainability and scalability remain limited by resource intensity and variable learner preparation. Future research should address long-term knowledge retention, equity across contexts, and cost-effectiveness to guide broader adoption.

## Introduction

Diabetes mellitus is a leading cause of morbidity and mortality worldwide, affecting more than 500 million people, with prevalence projected to rise further in the coming decades [1,2]. The global burden is substantial, with annual expenditure exceeding US$ 960 billion and rising [1,2]. Diabetes is also a major driver of cardiovascular disease, kidney failure, blindness, and lower-limb amputations, placing a profound strain on patients, families, and health systems alike. Effective management therefore requires not only biomedical knowledge but also clinical reasoning, communication, and sustained patient support. For pre-licensure health professions students including medical, nursing, and pharmacy learners, training must extend beyond factual recall to the cultivation of applied, patient-centred competence [3]. Because diabetes integrates complex pathophysiology, long-term management strategies, and sensitive communication needs, it provides a particularly rich context for evaluating active learning strategies that emphasise application, reflection, and practice [4].

Traditional didactic lectures remain widely used but consistently underperform compared with active pedagogies in promoting higher-order outcomes such as problem solving, durable retention, and authentic clinical performance [5–7]. In addition to lectures, diabetes education in many settings has traditionally relied on textbook reading, instructor-led seminars, and case-based discussions. While these approaches can provide foundational knowledge, they are often limited in fostering clinical reasoning, decision-making under uncertainty, and long-term skill retention. By contrast, flipped and blended classrooms, simulation-based training, gamification, virtual and technology-enhanced methods, and experiential learning activities have increasingly been incorporated into diabetes education [8,9]. The COVID-19 pandemic further accelerated the adoption of digital and hybrid models, highlighting both the promise of these approaches and persistent challenges such as uneven implementation quality, limited access to digital tools, inequities in learner readiness, and uncertain scalability [10].

Although systematic reviews in medical education have established the general effectiveness of active learning, none have focused specifically on diabetes education across health professions students [11–13]. Equity considerations are increasingly central to diabetes education, as evidence demonstrates that active learning effectiveness may vary by learner access to technology, digital literacy, language proficiency, and prior educational preparation. Underserved populations face documented barriers including limited internet connectivity, inadequate device access, cultural misalignment of educational materials, and reduced exposure to technology-enhanced learning in prior education [12,14]. These barriers affect not only patient education but also health professions students from disadvantaged backgrounds who may struggle with resource-intensive active learning modalities.This represents an important evidence gap, as the literature remains fragmented across disciplines and methodologies, long-term outcomes such as knowledge retention are rarely measured, and differences between professional groups have not been systematically synthesised. Moreover, existing reviews have emphasised educational effectiveness but have not systematically addressed feasibility, resource intensity, or equity factors that increasingly shape curriculum choices in resource-constrained environments.

A narrative synthesis is well suited for addressing this gap, as it can accommodate the heterogeneity of study designs, intervention types, and outcome measures while integrating both effectiveness and implementation perspectives. Theoretical frameworks further strengthen the rationale for this synthesis. Constructivism supports flipped classrooms by emphasising learner-centred construction of knowledge through pre-class preparation and in-class application [15,16]. Cognitive Load Theory underpins blended models that segment content across phases to optimise cognitive processing [17]. Experiential Learning Theory is operationalized in simulation and skills labs, where learners cycle through concrete experience, reflection, and application [18]. Social Learning Theory explains the value of modelling and feedback inherent in standardised patient encounters and team-based activities [19]. Finally, Self-Determination Theory clarifies how gamification enhances learner motivation through autonomy, competence, and relatedness [20]. Explicitly linking these theories to methods underscores why diabetes education is an ideal domain for testing diverse active strategies.

This review examines active learning strategies in diabetes education for pre-licensure medical, nursing, and pharmacy students. Other professional programmes such as nutrition, psychology, or social work were outside the scope of this review because of differing educational pathways and licensure structures. We acknowledge that nutrition professionals, psychologists, social workers, and other allied health disciplines provide critical diabetes self-management education and behavioural support, their educational programmes differ in structure, duration, competency frameworks, and assessment approaches. These differences would require separate analytical frameworks to ensure valid comparisons. We recognise this as a limitation and discuss implications for interprofessional education in our conclusions.

Our synthesis summarises intervention characteristics and educational outcomes, compares patterns across methods and learner groups, and evaluates feasibility and scalability considerations including cost and equity implications. By integrating outcomes with implementation factors, this review provides guidance for educators and curriculum designers on pedagogies that are not only effective but also practical and sustainable within contemporary health professions education.

## Methods

### Study design and rationale

This narrative review synthesises evidence on active learning approaches in diabetes education for health professions students, following established guidelines for systematic narrative synthesis [21]. We selected a narrative synthesis approach over systematic review with meta-analysis due to anticipated heterogeneity in educational interventions, outcome measures, and study designs across this rapidly evolving field. This methodology allows for comprehensive examination of diverse active learning modalities while maintaining analytical rigour appropriate for complex educational interventions.

### Search strategy and information source

We searched PubMed, Embase, Scopus, Web of Science, and Cochrane Library for studies published from 2010 to 2025 combining terms for active learning methods, health professions students, and diabetes education. We included peer-reviewed studies of active learning interventions in diabetes education for pre-licensure medical, nursing, or pharmacy learners that reported quantitative outcomes. Pre-licensure refers to learners enroled in degree programmes prior to independent clinical practice eligibility, regardless of undergraduate or graduate degree designation. Studies were screened for eligibility and data extracted on intervention characteristics, outcomes, and implementation factors. Quality was assessed descriptively based on study design, sample size, and outcome measurement rigour [22].

The search was limited to 2010 to 2025. The lower bound was selected to capture the contemporary period in which active and technology-enhanced learning approaches gained emphasis in health professions education following major curriculum reform initiatives and expansion of digital learning infrastructure during this period [23,24]. Limiting the window ensured relevance to current pedagogical practice while maintaining a sufficient time horizon for synthesis.

Our Boolean search strategy combined three concept groups using the AND operator: (1) active learning methods including ‘flipped classroom,’ ‘blended learning,’ ‘gamification,’ ‘simulation-based learning,’ ‘virtual reality (VR),’ ‘problem-based learning,’ ‘case-based learning,’ and ‘technology-enhanced learning’; (2) health professions learners including ‘medical students,’ ‘nursing students,’ ‘pharmacy students,’ and ‘health professions education’; and (3) diabetes-related content including ‘diabetes mellitus,’ ‘diabetes education,’ ‘endocrinology,’ and ‘chronic disease management.’ Reference lists of included studies underwent manual screening to identify additional relevant articles not captured by electronic searches [25].

### Study selection and eligibility criteria

Two reviewers independently screened all titles and abstracts, followed by full-text review of potentially eligible studies [26]. Disagreements were resolved through discussion, with third-party arbitration when consensus could not be reached. Studies were included if they met the following criteria: peer-reviewed publications in English; pre-licensure learners included as participants; educational interventions employing active learning methods defined as pedagogical approaches requiring learners to engage meaningfully with content through analysis, synthesis, or application rather than passive information reception [7]; diabetes-related educational content; and quantitative or mixed-methods designs reporting educational outcomes such as knowledge acquisition, clinical skills development, or learner satisfaction. We excluded studies focusing solely on patient education, postgraduate specialist training programmes, purely qualitative studies without quantitative outcome measures, and conference abstracts without full peer review. Studies involving other professional groups such as nutrition, psychology, social work, allied health or interprofessional cohorts were excluded when separate results for core medical, nursing, or pharmacy learners could not be abstracted due to lack of comparable competencies, licensure pathways, or curricular sequencing.

Exclusion criteria also included lack of diabetes-specific educational content, absence of an identifiable active learning intervention, unextractable outcome data, duplicate reports, and incomplete full-text availability. Exclusion categories and corresponding counts are summarised in the PRISMA flow diagram and described narratively in the Results section.

### Data extraction and study characteristics

A standardised data extraction form was developed a priori, pilot-tested on five studies, and refined before full implementation. Two reviewers independently extracted study characteristics including design, setting, and sample size; participant details including profession and academic level; intervention features including active learning methods employed, technology platforms used, duration, and diabetes content covered; implementation factors including faculty time, resource needs, and reported challenges; and outcome measures including assessment methods and results. Extraction discrepancies were resolved through discussion and re-examination of source materials. Inter-rater reliability demonstrated substantial agreement [27].

### Quality assessment

Study quality was assessed using the Medical Education Research Study Quality Instrument (MERSQI), an 18-point validated scale designed specifically for medical education research that evaluates study design, sampling methods, data type, validity of evaluation instruments, data analysis, and outcomes [28]. We assessed study quality descriptively, noting design features (randomisation, control groups, blinding), sample characteristics (size, single vs. multi-site), and measurement rigour (validated instruments, objective vs. self-report outcomes).

### Data synthesis and analysis

We employed thematic narrative synthesis organised around three key domains: learning effectiveness (knowledge gains, skill acquisition, competency development), learner engagement (satisfaction, participation, preference over traditional methods), and implementation feasibility (resource requirements, scalability, sustainability). Studies were initially grouped by primary active learning method and target population, then synthesised across these categorical boundaries to identify overarching patterns and themes.

To make explicit how conclusions were derived, two reviewers independently coded each included study into predefined outcome domains: knowledge, skills/competency (including clinical reasoning/OSCE-type outcomes where applicable), attitudes/self-efficacy, learner engagement/satisfaction, and implementation/feasibility. For effectiveness outcomes, we extracted the quantitative results as reported (pre–post change and/or between-group differences) and summarised findings using direction-of-effect coding (improved/no meaningful change/worsened). We then identified cross-study patterns by examining consistency within outcome domains and prioritising conclusions supported by multiple studies and/or controlled designs.

Implementation data (e.g., faculty time, cost/resource needs, technology requirements, scalability barriers, and equity-related considerations when reported) were extracted using the same structured form and synthesised alongside effectiveness outcomes to support practice-relevant conclusions. Any disagreements in coding or interpretation were resolved by discussion and, when needed, third-reviewer arbitration.

## Results

**Figure 1.**
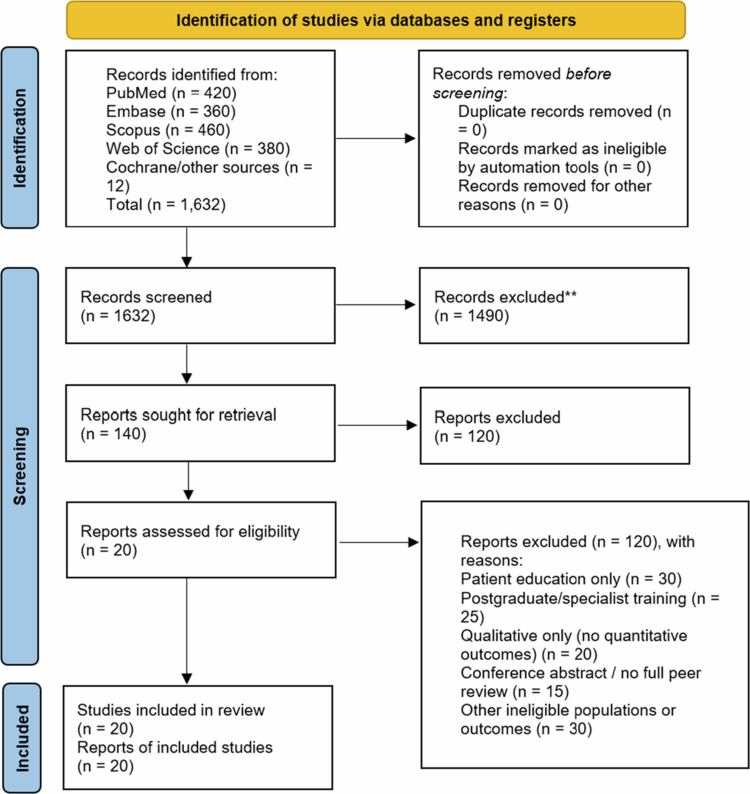
PRISMA flow diagram of study selection.

As shown in Figure 1, a total of 1,632 records were identified across PubMed (*n* = 420), Embase (*n* = 360), Scopus (*n* = 460), Web of Science (*n* = 380), and Cochrane/other sources (*n* = 12). The full text of 140 reports was assessed for eligibility, of which 120 were excluded. Ultimately, 20 studies met the eligibility criteria and were included in the final synthesis.

As shown in Table 1, the 20 included studies reported outcomes across key educational domains, most commonly knowledge acquisition (*n* = 15) and skills or OSCE-based performance (*n* = 7), followed by engagement or self-efficacy (*n* = 6) and learner satisfaction or acceptability (*n* = 5). Collectively, these studies enroled 1,854 participants, including medical students (*n* = 740), nursing students (*n* = 700), and pharmacy students (*n* = 414), with sample sizes ranging from 22 to 196 participants. Study designs comprised 10 randomised controlled trials, 3 quasi-experimental studies, and 7 single-group pre-post evaluations. Interventions primarily evaluated active learning approaches, including simulation and virtual modalities, gamification, flipped or blended formats, and technology-enhanced experiential activities, compared with didactic lectures, case-based instruction, or usual curriculum. Detailed study characteristics and intervention descriptors are provided in Supplementary Table S1.

**Table 1.** Educational outcomes reported across included studies, organised by primary outcome domain (*n* = 20).

Outcome domain	Strategy subtype	No. of studies	Direction	Representative studies
**Knowledge acquisition**	Simulation/VR/virtual simulation	5	↑	[29,30,31]
	Gamification (escape room/board/serious game)	6	↑	[32–34]
	Flipped/blended/intensive day	4	mixed	[9,35,36]
	Device-based experiential (e.g., CGM wear + counselling)	1	↑	[37]
**Clinical skills/OSCE/performance**	Simulation/SP/procedural practice	5	↑	[30,38,39]
	OSCE/structured performance assessment	2	↑	[35,40]
	Skills videos/demonstration-guided practice	1	↑	[41]
	Counselling skills	1	↑	[37]
	Skill-specific structured technique framework	1	↑	[42]
**Confidence/self-efficacy/attitudes/clinical thinking**	Simulation/VR/virtual care	3	↑	[29,31,40]
	Gamification	2	↑	[34,43]
	Case-based interactive/structured active sessions	3	↑	[44–46]
	Experiential device-based learning	1	↑	[37]
**Engagement/motivation/flow**	Gamification/serious game	2	↑	[34,43]
	Flipped learning	1	↑	[36]
	Interactive case-based/active learning	1	↑	[44]
**Satisfaction/acceptability**	Gamification	2	↑	[32,33]
	Flipped/blended learning	2	↑	[9,35]
	Simulation	1	↑	[38]

**Note:** Studies may be listed under more than one outcome domain when multiple outcomes were reported. Detailed study characteristics (e.g., design, country, learner level, intervention details) are provided in Supplementary Table S1.

Table 2 summarises outcomes across strategies. Simulation (9 studies) consistently improved knowledge, skills, and self-efficacy, particularly when debriefing and progressive case complexity were included; low-fidelity or lecture-linked designs showed weaker effects. Gamification (6 studies) improved engagement and learning, with the strongest results in board and escape games that emphasised teamwork; digital games yielded smaller gains due to usability and interaction limits. Flipped/blended learning (4 studies) produced mixed results, largely dependent on student preparation, with positive effects only when combined with experiential practice [35]. Technology-enhanced experiential methods (2 studies) improved knowledge and self-efficacy through authentic tasks such as CGM wear or virtual care, though scalability was constrained by cost and resources.

**Table 2.** Synthesis of Active Learning Outcomes and Theory-Driven Explanations.

Strategy	Studies (*n*, participants)	Outcome Direction	Core Theoretical Explanations
**Simulation (9)**	[9,29–31,38–40,45,46]	Mostly positive (knowledge, skills, self-efficacy). Gibbs modest; Moxley mixed	**Experiential Learning**: Structured debriefing completed full cycle (sim → reflection → conceptualisation → application). **Cognitive Load**: Progressive case complexity (e.g., vitals before insulin titration) reduced overload; mixed results when lecture + sim delivered simultaneously (Moxley). **Social Learning**: Faculty modelling supported transfer. **Self-Determination**: Safe space for failure (VR, HFS) promoted competence/confidence.
**Gamification (6)**	[32–34,42,43,47]	Positive overall; strongest in board/escape games (large knowledge gains); weaker in digital (modest gains)	**Social Learning**: Peer reasoning visible in physical games; limited in digital formats. **Self-Determination**: Relatedness and team cohesion drove engagement in escape rooms; digital play lacked this. **Cognitive Load**: Simple game rules reduced extraneous load; digital formats added interface/navigation load. Escape rooms balanced time pressure (load) with teamwork (distributed reasoning), yielding high motivation and immediate feedback.
**Flipped/Blended (4)**	[9,35,36,45]	Mixed: Farahani positive (knowledge + OSCE); others showed little/no advantage	**Constructivism**: Success depends on students preparing before class; non-compliance (Wu, Patnaik) undermined active learning. **Cognitive Load**: Pre-class videos often dense, overloading intrinsic load; segmentation absent. **Self-Determination**: Lack of adaptive scaffolding and autonomy limited motivation. **Experiential Learning**: Farahani’s integration of OSCE created authentic practice, completing the experiential cycle (e-learning → discussion → OSCE).
**Technology-Enhanced Experiential (2)**	[37,40]	Both positive (knowledge, counselling, self-efficacy)	**Experiential Learning**: Authentic experience central, Folz (wearing CGM, then debrief), Xu (virtual patient care, iterative cases). **Reflection & Application**: Students reviewed personal or simulated data before applying in counselling/SP encounters. **Social Learning**: Peer collaboration (Xu) and standardised patient interactions (Folz) reinforced professional modelling. **Feasibility constraints**: Cost of CGM devices and multi-week delivery limit scalability despite strong outcomes.

**Figure 2.**
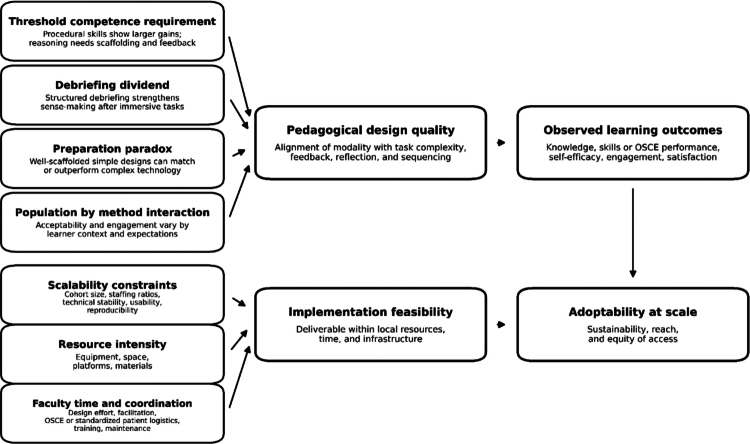
Conceptual framework summarising mechanisms and feasibility factors that may explain variability in learning outcomes and adoptability across active learning interventions.

Table 3 summarises cross-cutting explanatory themes, and Figure 2 presents these themes as a conceptual framework linking pedagogical mechanisms and implementation feasibility to observed learning outcomes and adoptability. Four recurring themes emerged.

**Table 3.** Implementation considerations and cross-cutting patterns that explain variability in outcomes of active learning interventions for diabetes education.

Domain	Synthesis element	What was observed across studies	Interpretation and practical implication
Cross-cutting patterns explaining heterogeneity	Debriefing dividend	Simulation and virtual reality interventions that included structured debriefing showed more consistently positive effects than those without debriefing.	Debriefing likely strengthens learning by completing the experiential cycle and supporting sense-making after complex tasks. For higher-fidelity approaches, debriefing should be treated as a core component rather than an optional add-on.
	Preparation paradox	Lower-preparation, simpler designs sometimes outperformed complex, technology-heavy interventions.	Effectiveness appears to depend more on instructional sequencing and minimising unnecessary complexity than on preparation time alone. When resources are constrained, well-scaffolded low-tech approaches may yield strong value.
	Population by method interaction	Nursing and pharmacy learners tended to report higher engagement and satisfaction with active learning, while results among medical learners were more mixed.	Differences may reflect fit between pedagogy and learner expectations or norms rather than discipline alone. Implementation should consider learner context and provide explicit rationale, expectations, and structure to support buy-in.
	Threshold competence requirement	Very large effects were most common for lower-complexity procedural skills, whereas higher-order reasoning showed smaller or mixed gains unless paired with scaffolding and debriefing.	Match method to task complexity. Procedural competencies may respond to focused skills practice, while clinical reasoning benefits from progressive case complexity, guided reflection, and feedback.
Implementation and scalability synthesis by strategy	Board games and low-resource gamification	Typically low resource intensity and high scalability; primary burden is initial design time and space for facilitation.	Strong option for broad uptake where budgets are limited. Emphasise clear rules, teamwork, and short feedback loops to maintain engagement while keeping delivery costs low.
	Escape rooms	High upfront planning burden, including reports of extended development timelines, but scalable once established; coordination complexity is common.	Consider as a ‘build once, run many times’ model. Standardised materials and clear facilitator guides improve reproducibility and reduce variability across implementations.
	Flipped and blended learning	Often low to medium resource intensity and moderate to high scalability; a recurrent challenge is inconsistent student preparation and time-intensive design of pre-class materials.	Pre-class compliance is a primary determinant of success. Use short segmented materials, low-stakes accountability, and in-class application activities that clearly reward preparation.
	Blended e-learning with OSCE or standardised patients	Moderate resource intensity; logistical complexity includes standardised patient training and OSCE coordination; scalability is typically moderate.	Best suited when performance assessment is a priority. Standardisation of raters and scenarios helps reduce variability and protects validity of observed skill gains.
	High-fidelity simulation and device-based experiential learning	Highest resource intensity due to equipment, laboratory needs, and faculty oversight; scalability constrained despite strong educational effects.	Reserve for high-stakes skills or complex decision-making where realism and deliberate practice are essential. Use structured debriefing and progressive complexity to maximise return on investment.
	Virtual simulation and custom digital platforms	Higher development costs and maintenance needs, but potential for strong scalability once built; usability and interface issues are recurring barriers.	Useful when institutions want scale without physical simulation constraints. Early usability testing and iterative updates are critical to avoid interface-related learning friction.
	Digital games requiring intensive facilitation	High ongoing personnel demands can limit scalability, even when engagement outcomes are strong.	Consider redesign toward small-group or peer-facilitated formats to reduce one-to-one ratios, or position as a targeted enrichment activity rather than a required large-cohort intervention.

Note: This table synthesises cross-cutting patterns and implementation considerations reported across studies to support interpretation of heterogeneity and real-world feasibility. Abbreviation: OSCE = Objective Structured Clinical Examination.

First, outcomes were more consistently favourable when immersive activities, particularly simulation and virtual modalities, were paired with structured debriefing, underscoring the importance of guided reflection in consolidating learning after complex tasks. Second, a ‘preparation paradox’ was evident in that simpler, lower-preparation designs sometimes yielded outcomes comparable to, or stronger than, more technology-intensive approaches, suggesting that instructional sequencing and cognitive manageability may be as influential as modality.

Third, engagement and satisfaction appeared to vary by learner group, with nursing and pharmacy cohorts more consistently reporting positive experiences than medical cohorts, indicating that learner expectations and contextual fit may shape acceptability and, in turn, effectiveness. Finally, effects were largest for lower-complexity procedural competencies, whereas gains in higher-order clinical reasoning were more variable and appeared to require deliberate scaffolding and feedback. In parallel, implementation feasibility differed markedly by strategy.

Low-resource gamified approaches offered high scalability with limited infrastructure demands, whereas escape rooms and high-fidelity simulation required substantial upfront development, coordination, and faculty time. Virtual platforms presented high initial design and maintenance costs but offered potential for scale once established, contingent on usability and technical stability.

**Figure 3.**
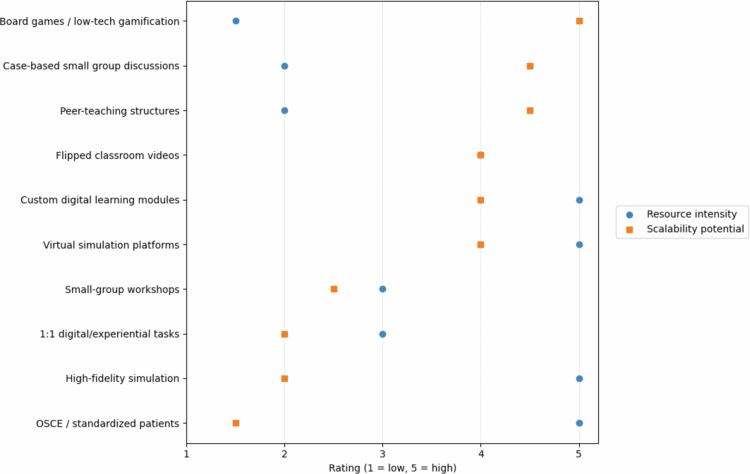
Resource intensity and scalability potential of active learning strategies in diabetes education. The horizontal axis represents ratings from 1 (low) to 5 (high). Circles indicate resource intensity, defined by requirements for infrastructure, technology, faculty time, and coordination. Squares indicate scalability potential, defined by the feasibility of implementation across larger cohorts and institutional contexts. Low-resource strategies such as board games, case-based discussions, and peer-teaching structures demonstrate high scalability, whereas high-fidelity simulation and OSCE/standardized patient approaches show high resource demands and lower scalability. Virtual simulation platforms and custom digital modules exhibit higher scalability following initial development despite moderate to high resource investment.

**Figure 4.**
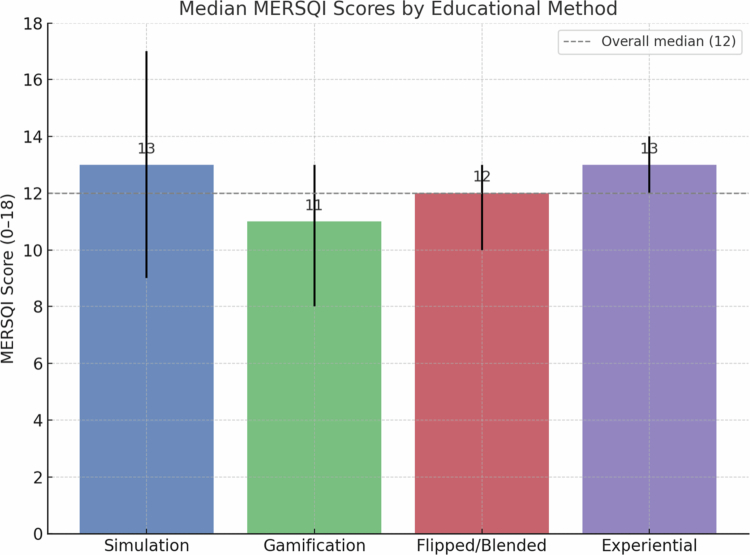
Median MERSQI scores by educational method, with error bars showing the observed ranges.

Analysis of the included studies revealed meaningful variation in resource requirements and scalability across active learning strategies. To support interpretation and future implementation planning, we visually summarised feasibility characteristics using independent ratings of resource intensity and scalability potential. As shown in Figure 3, low-tech gamification, case discussions, and peer-teaching approaches tended to require fewer resources while remaining scalable across institution types. In contrast, high-fidelity simulation, OSCE-based approaches, and device-dependent experiential activities demonstrated lower scalability due to personnel and infrastructure demands. Notably, virtual simulation and digital modules showed relatively high scalability despite greater initial resource investment, highlighting opportunities for reusable technology-enabled design (Figure 4).

The methodological quality of included studies, assessed using the Medical Education Research Study Quality Instrument (MERSQI), was moderate overall. Median scores ranged from 11 to 13 out of 18, with an overall median of 12 (Figure 3).

## Discussion

This narrative synthesis examined active learning approaches in diabetes education across 20 studies involving 1,854 health professions students. Overall, the evidence suggests that active learning strategies outperform traditional didactic instruction, although effectiveness varied across methods and learner populations. Simulation-based learning demonstrated the most consistent benefits, while virtual and other technology-enhanced approaches and gamification also showed promise. Flipped classrooms yielded mixed outcomes despite their popularity. Because interventions and outcome measures varied across studies, we emphasise cross-study patterns rather than single-study findings. Interpretations consider study quality and design features.

### Effectiveness by educational method

In our review, high-fidelity simulation consistently produced large improvements in knowledge, skills, and self-confidence for diabetes education. Studies reported significant gains in satisfaction and confidence after simulation-based DKA training compared to clinical placement [29], very large immediate and sustained knowledge improvements in hypoglycemia management [38], enhanced diabetic foot examination skills using both high-fidelity simulators and standardised patients [39], and improved knowledge and confidence when simulation was integrated into didactic instruction [46]. Reviews and meta-analyses confirm these benefits, showing moderate to large learning gains and highlighting simulation as one of the most reliable active learning methods in health professions education [48–50].

At the same time, evidence suggests simulation may not always be optimal for factual recall. For example, one study [30] found students in case-based learning scored higher on written knowledge tests, although simulation participants performed better in clinical skills. Taken together, these results suggest that simulation is particularly effective for applied competence and confidence building, whereas case-based methods may reinforce theoretical knowledge.

Simulation appears superior for applied performance because it provides experiential, context-rich practice that mirrors real clinical encounters. Learners actively integrate knowledge with psychomotor skills, decision-making, and teamwork in a safe environment where mistakes can be corrected through debriefing and feedback. This immersion supports transfer of skills to authentic patient care. These outcomes are difficult to achieve through didactic or purely cognitive approaches.

Overall, both our findings and the external literature suggest simulation is best applied when the primary educational goal is clinical performance transfer and confidence building, while case-based or blended strategies may complement knowledge acquisition.

Virtual simulation and other technology-enhanced approaches show consistent promise for diabetes and related clinical education. A large randomised trial in the UK reported that desktop-based VR modules improved knowledge and clinical performance compared with traditional case studies [31]. Reviews of virtual simulation in nursing education conclude that it enhances knowledge, practice proficiency, skills retention, and satisfaction, although gains in critical thinking are less consistent [51–53]. These findings align with experiential learning theory, which supports learning through application and reflection.

However, digital equity remains a concern in lower-resource settings [54]. Analyses also suggest that while virtual methods can match or exceed high-fidelity simulation in accessibility and cost-effectiveness, they may not always deliver superior confidence or long-term retention [52]. This pattern suggests virtual simulation is an effective and scalable adjunct, although structured debriefing remains critical.

Gamification strategies produced largely positive results, though effectiveness differed by format. Board games such as Diabe-teach [47] and Candy Gland [43] consistently improved knowledge, confidence, and engagement. By combining repetition, peer interaction, and low cognitive load, board games appear to balance motivation and comprehension. This aligns with self-determination theory, where autonomy, competence, and relatedness enhance intrinsic motivation.

Digital gamified tools, including escape rooms and interactive patient cases, demonstrated substantial short-term gains in knowledge and satisfaction [32,33]. Scoping reviews caution that poorly designed digital gamification can introduce unnecessary complexity, limiting knowledge transfer (Subhash and Cudney, 2018). Physical games may reduce extraneous cognitive load through social interaction, which could explain more consistent results.

Flipped and blended learning approaches showed inconsistent effects. Only one of three studies demonstrated clear knowledge benefits [10,35,36], and satisfaction remained high even when learning outcomes were modest. This gap between learner preference and objective outcomes highlights potential misalignment between perceived and actual learning.

### Professional context and population differences

Nursing students consistently showed greater response across all active learning methods compared to medical or pharmacy students. This pattern may reflect nursing education's traditional emphasis on procedural competence and collaborative learning, making active approaches more naturally aligned with existing pedagogical culture. Medical students showed more variable responses, particularly to flipped classroom approaches. This may reflect expectations for didactic instruction or assessment pressures that favour memorisation. Pharmacy students demonstrated strong responses to hands-on experiential learning but less consistent benefits from cognitive interventions. These findings suggest that tactile or procedure-oriented strategies may be particularly valuable for medication management competencies.

Although our synthesis focused on pre-licensure learners in medicine, nursing, and pharmacy programmes, we acknowledge that other health professions students such as nutrition, psychology, social work, and allied disciplines play essential roles in diabetes care and education. These disciplines contribute meaningfully to interprofessional diabetes management, and we plan future work that will expand the synthesis to include their experiences and contributions to active learning approaches.

### Implementation science considerations

High-fidelity simulation, despite strong educational outcomes, requires substantial initial investment. Freestanding simulation centres report startup costs between US$200,000 and US$1 million and individual mannequins costing tens of thousands [55,56]. Faculty preparation time varies widely. Virtual simulation programmes frequently require extensive scenario development, debriefing, and technology training [51], whereas simpler gamified methods require less setup. Equity considerations are increasingly important, as underserved populations face barriers in device access, digital literacy, and cultural or language fit [12,14].

### Evidence quality and limitations

Evidence quality was assessed using MERSQI scores. Most included studies had methodological limitations including small sample sizes, single-institution designs, and short follow-up periods. These limitations restrict precision and generalisability. A key limitation is the absence of long-term follow-up, which limits conclusions about durability of learning. No study examined patient outcomes or longer-term clinical performance links.

### Theoretical implications

The differential effectiveness patterns support cognitive load theory predictions about optimal learning conditions. Methods that segmented complex diabetes content across multiple modalities consistently outperformed single-mode interventions. Social learning theory's emphasis on modelling and feedback appears relevant for simulation and standardised patient encounters. The consistently high satisfaction with collaborative approaches suggests a motivational role for relatedness as emphasised in self-determination theory.

### Practice implications

These findings suggest a strategic approach prioritising simulation and gamification over popular but inconsistently effective flipped classroom models. Institutions with limited resources might implement low-cost physical games before investing in expensive simulation equipment. Diabetes education appears to benefit from combining foundational knowledge transmission with high-impact active learning experiences targeted to specific competencies. Faculty development should emphasise facilitation and debriefing skills, as these human elements appear central to success across modalities.

### Future research priorities

Longitudinal effectiveness studies represent the most critical research gap. Understanding which approaches produce durable retention would inform curriculum investment decisions. Multi-institutional studies could address sample size limitations and explore contextual moderators of effectiveness. Cost-effectiveness analyses could guide resource allocation decisions. Patient outcome linkage studies could determine whether educational innovations improve clinical practice and quality of care.

### Limitations

This synthesis has several important limitations that warrant consideration. First, the small number of included studies (*n* = 20) with generally modest sample sizes limits statistical precision and the strength of conclusions that can be drawn. Single-institution designs dominate the literature, restricting generalisability across diverse educational contexts, resource settings, and student populations.

Second, equity and access considerations represent a critical gap in the current evidence base. While we examined implementation feasibility, few included studies explicitly reported on equity-related barriers or disaggregated outcomes by student socioeconomic status, race/ethnicity, first-generation college status, or technology access. This limits our ability to determine whether active learning approaches benefit all students equally or inadvertently advantage those with greater resources, prior technology exposure, or flexible schedules. Digital divide concerns are particularly salient for technology-enhanced and flipped classroom approaches that require personal devices, reliable internet, and self-directed digital literacy—resources not universally available to students from underserved backgrounds [54]. Similarly, high-fidelity simulation and device-based experiential learning may create geographic or financial barriers for students at under-resourced institutions or those balancing education with employment and caregiving responsibilities. Future research must explicitly examine differential effectiveness across student subgroups and identify design modifications that promote equitable access and outcomes.

Third, professional representation was uneven, with nursing students comprising the largest proportion of participants and some studies combining professional groups without separate outcome reporting. Differences in curricular structure, assessment methods, and competency frameworks across medicine, nursing, and pharmacy may moderate intervention effects in ways we could not fully disentangle. As noted previously, the exclusion of nutrition, psychology, social work, and allied health professions limits applicability to interprofessional diabetes education, though this decision reflected the need for comparable competency frameworks and assessment approaches.

Fourth, the absence of long-term follow-up represents a major evidence gap, as no included studies examined knowledge retention beyond the immediate post-intervention period or assessed whether educational gains translated to improved clinical performance or patient outcomes. Without such data, we cannot determine whether observed benefits endure or influence practice behaviour.

Fifth, the search was restricted to studies published from 2010 onward to reflect contemporary pedagogy following major curriculum reforms and digital learning infrastructure expansion. While this ensured relevance to current practice, it may have excluded earlier foundational studies. Additionally, restriction to English-language publications may have introduced cultural and geographic bias.

Finally, methodological quality varied substantially across studies, with many lacking adequate control groups, randomisation, blinding, or validated outcome measures. The predominance of self-reported outcomes (satisfaction, confidence) over objective performance assessments limits conclusions about actual competency development.

## Conclusions

This review highlights that technology-enhanced learning in diabetes education produces varied benefits across modalities. High-fidelity simulation consistently demonstrates strong effects on knowledge, skills, and confidence, although resource intensity remains a barrier. Virtual simulation extends these benefits with scalability and engagement, but requires institutional support and faculty preparation. Gamification approaches, particularly board games, show promise in enhancing motivation and reinforcing knowledge with minimal faculty preparation. Flipped and blended learning approaches display inconsistent effects on objective knowledge outcomes.

Implementation considerations are critical. Cost, institutional support, and equity issues require deliberate planning. Technology-dependent strategies may disadvantage learners without reliable access to devices or the internet. Integrative reviews emphasise that self-management education for underserved groups must account for contextual barriers and adapt interventions accordingly [12,14].

Overall, technology-enhanced approaches can enrich diabetes education, but no single method is universally superior. A balanced hybrid model using simulation for applied skills, gamification for engagement, and case-based or flipped approaches for conceptual reinforcement may offer beneficial learning outcomes while addressing the practical constraints of resource availability, equity, and sustainability.

## Supplementary Material

Supplementary Material Supplementary tables .docx

## Data Availability

The datasets (3rd party material) used and/or analysed during the current study are available from the corresponding authors on reasonable request.
