# Supplementary material for: Active learning in diabetes education for health professions students: a narrative synthesis of effectiveness and implementation considerations
Source: Med Educ Online. 2026 Apr 22;31(1):2656830. doi: 10.1080/10872981.2026.2656830 (PMC13103985; doi:10.1080/10872981.2026.2656830)
Supplement: Supplementary Material — Supplementary tables .docx [file ZMEO_A_2656830_SM3292.docx]

Supplementary Table S1. Characteristics of Included Studies

| **Author, Year** | **Design** | **Country** | **Population** | **Learner Stage** | **Intervention vs Comparator (Population, N)** | **Primary Outcome(s)** |
| --- | --- | --- | --- | --- | --- | --- |
| Wu et al., 2024 | RCT crossover | China | Medical students | 6th year (8-year program) | Flipped classroom (videos + presentations) vs lecture (Medical students, China, n=93) | Knowledge, Satisfaction |
| Farahani et al., 2020 | Pre-post single cohort | Germany | Undergraduate pharmacy students (8th semester) | Undergraduate | Blended e-learning + OSCE vs none (Pharmacy, Germany, n=58) | Knowledge, OSCE, Satisfaction |
| Kiles et al., 2021 | Single-group educational intervention (post-then-pre self-assessment survey) | USA | Pharmacy students (2nd year, PharmD program) | Undergraduate | Virtual CYOA case vs none (Pharmacy, USA, n=178) | Self-efficacy, Engagement |
| MacEwen et al., 2016 | Pre-post single-group pilot study | UK (University of Glasgow) | Undergraduate medical students (final clinical year) | Undergraduate | Acute Care Day (lectures + case tutorials) vs none (Medical, UK, n=196) | Knowledge, Attitudes |
| Eukel et al., 2017 | Pre-post study | USA | Pharmacy students | 3rd year professional (P3) | Diabetes escape room vs none (Pharmacy, USA, n=74) | Knowledge, Satisfaction |
| D’Souza et al., 2020 | RCT | Oman | Nursing students | Undergraduate critical care nursing | High-fidelity DKA simulation vs clinical placement (Nursing, Oman, n=140) | Knowledge, Skills |
| Dincer et al., 2020 | RCT | Turkey | Nursing students | 2nd year undergraduate | High-fidelity simulation vs lecture (Nursing, Turkey, n=61) | Knowledge, Satisfaction |
| Patnaik et al., 2025 | Quasi-experimental | India | Medical students | 8th semester | Flipped classroom vs lecture (Medical, India, n=130) | Knowledge, Engagement |
| Hibbert et al., 2013 | RCT | Australia | Medical students | 2nd year | Online skills videos vs standard revision (Medical, Australia, n=22) | Clinical skills |
| Inkaya et al., 2020 | RCT | Turkey | Nursing students | 4th year | Simulator vs standardized patients (Nursing, Turkey, n=42) | Clinical exam performance |
| Moxley et al., 2021 | Pre-post study | USA | Nursing students | Master's Entry to Practice | Simulation + didactic vs none (Nursing, USA, n=46) | Skills, Confidence |
| Singleton et al., 2021 | RCT | UK | Nursing students | 2nd year | VR simulation (hypoglycemia) vs paper case (Nursing, UK, n=171) | Knowledge, Self-efficacy |
| Gibbs et al., 2014 | Quasi-experimental | USA | Nursing students | 1st year associate degree | Low-fidelity HPS vs tabletop case (Nursing, USA, n=96) | Clinical performance |
| Xu et al., 2025 | Quasi-experimental | China | Nursing interns | Clinical internship (endocrinology) | Virtual diabetes care simulation vs face-to-face teaching (Nursing interns, China, n=69) | Knowledge, Skills |
| Shadan et al., 2025 | RCT | UAE | Medical students | Preclinical (Years 1-3) | “Diabe-teach” board game vs self-study (Medical, UAE, n=56) | Knowledge |
| Kavanaugh et al., 2020 | Cross-sectional pre-post | United States | Pharmacy students | Undergraduate | Team-based escape room vs none (Pharmacy, USA, n=124) | Knowledge, Satisfaction |
| Soylu et al., 2025 | Mixed-methods RCT | Turkey | Nursing students | Undergraduate (2nd year) | Six Hats vs Video vs Lecture (Nursing, Turkey, n=90) | Knowledge |
| Folz et al., 2025 | Multi-institutional pragmatic RCT | United States | Pharmacy students | Undergraduate (P2-P3) | CGM wear + lecture/demo vs lecture/demo (Pharmacy, USA, n=63) | Knowledge, Counseling skills |
| Twist & Ragsdale, 2022 | Pre-post intervention study | United States | Medical students | Undergraduate (2nd year) | “Candy Gland” board game vs none (Medical, USA, n=99) | Knowledge |
| Tan et al., 2024 | Mixed-methods open-label superiority RCT | Singapore | Medical students | Undergraduate (3rd-5th year) | Digital serious game ± facilitator (Medical, Singapore, n=48) | Knowledge, Engagement |

Supplementary Table S2: Key outcomes and effects

| **Author, Year** | **Primary Outcomes** | **Key Results (Baseline → Post → Follow-up where available)** | **Significance** | **Student Satisfaction (%)** |
| --- | --- | --- | --- | --- |
| Wu et al., 2024 | Knowledge, Satisfaction | DR knowledge ↑ (NS); satisfaction is high | Not reported | 93% satisfied; 88% preferred flipped |
| Farahani et al., 2020 | Knowledge, OSCE, Satisfaction | Knowledge 43% → 78%; OSCE 35% → 69%; Self-assess 43% → 62% | p<0.001 | 72-85% positive |
| Kiles et al., 2021 | Confidence, Engagement | Self-rated skills improved (all p<0.001) | NA (self-report Likert) | 84% engaging; 87% improved understanding |
| MacEwen et al., 2016 | Knowledge, Confidence | MCQ 27% → 47%; Confidence VAS ↑25 points | p<0.001 | NR |
| Eukel et al., 2017 | Knowledge, Satisfaction | Knowledge 56% → 81% | Not reported | Mean 4.1/5 |
| D’Souza et al., 2020 | Critical thinking, Self-confidence | Satisfaction 23 → 25; Confidence 33 → 36 | Not reported | 4.2/5 |
| Dincer et al., 2020 | Knowledge, Satisfaction | Knowledge 73 → 95 (post), 88 (1-mo FU) | Not reported | 76% satisfied |
| Patnaik et al., 2025 | Knowledge, Engagement | Knowledge 6.0 → 8.0 | Not reported | 78% positive |
| Hibbert et al., 2013 | Clinical skills | Competency 20-40% vs 83-92% with videos | Not reported | 92% strongly agreed videos helpful |
| Inkaya et al., 2020 | Skills, Knowledge | Skills ↑ large (HFS/SP both d >3.0) | Not reported | Positive feedback |
| Moxley et al., 2021 | Knowledge, Confidence | Confidence d = 1.7; Knowledge d = 1.3 | Not reported | 98% found relevant |
| Singleton et al., 2021 | Knowledge, Self-efficacy | VR > paper case, all p<0.001 | Not reported | Positive qualitative |
| Gibbs et al., 2014 | Knowledge, Clinical skills | Mixed: case > HPS for knowledge; HPS > case for skills | Not reported | HPS group rated higher |
| Xu et al., 2025 | Self-directed, Clinical thinking | Self-directed 97 → 105; Clinical thinking 85 → 100 | Not reported | 8.4/10 |
| Shadan et al., 2025 | Knowledge, Confidence | Post-test 82 vs 69 (game vs control) | Not reported | 100% preferred game |
| Kavanaugh et al., 2020 | Knowledge, Satisfaction | Knowledge ↑ 20-25 points | p<0.05-0.001 | 4.2-4.6/5 |
| Soylu et al., 2025 | Insulin injection skills | Six Hats > Video > Control at 3 mo | β=5.4-10.9, p<0.001 | Positive themes |
| Folz et al., 2025 | Counseling skills, Confidence | Counseling ↑ 5% (p=0.046); Confidence ↑0.6 (p=0.03) | Small-moderate | Effective (Likert 1.8-2.0/2) |
| Twist & Ragsdale, 2022 | Knowledge, Confidence | Knowledge 7.3 → 8.0; Confidence ↑ across domains | p<0.001 | 91-96% positive |
| Tan et al., 2024 | Flow, Engagement | Flow d = 0.63; Absorption d = 0.75 | Significant | High engagement |

**Assessment scales: **Skills (1-5 scale), ***Confidence (1-7 scale), ***Competency (1-5 scale)*

Supplementary Table S3. Implementation factors, scalability, and reported challenges of active learning interventions in diabetes education for health professions students.

| **Author, Year** | **Technology Platform** | **Faculty Time** | **Resource Intensity** | **Scalability Rating** | **Reported Challenges** |
| --- | --- | --- | --- | --- | --- |
| Wu, 2024 | Pre-recorded videos + PCs | Prep videos (NR) | Low-Medium | High | Inadequate student prep time |
| Farahani, 2020 | Moodle LMS + OSCE | 2 trainers + SPs (~16h total) | Medium | Medium | SP training variability; OSCE logistics |
| Kiles, 2021 | Zoom + survey platform | 1 faculty + TAs | Low | High | Variability in TA facilitation |
| MacEwen, 2016 | Face-to-face lectures/tutorials | Multiple lecturers | Moderate | Medium | Many facilitators required |
| Eukel, 2017 | iPads + Google Hangout | 20h setup; 2h delivery | Low | High | Coordination complexity |
| D’Souza, 2020 | High-fidelity SIM lab | 40h over 14 wks | High | Medium | Resource-intensive; lab required |
| Dincer, 2020 | High-fidelity simulator | NR | Medium-High | Medium | Ignores individual learning styles |
| Patnaik, 2025 | Videos + articles | High prep time | Medium | Medium | 15% students skipped pre-class work |
| Hibbert, 2013 | Downloadable videos | Video prep (NR) | Medium | High | Tech barriers (downloads, devices) |
| Inkaya, 2020 | Simulator + SPs | Scenario + SP training | High | Medium | Small sample; resource heavy |
| Moxley et al., 2021 | Simulation + didactic | NR | Medium | High | Limited clinical opportunities |
| Singleton, 2021 | Desktop VR (Unity 3D) | High (VR creation) | Medium-High | High | Short-term only; motion sickness rare |
| Gibbs et al., 2014 | Low-fidelity mannequin | High (training + dev.) | Medium-High | Medium | Biased eval tool; resource burden |
| Xu, 2024 | Custom 3D virtual sim app | High (app dev.) | High | Medium-High | Interface usability, update needs |
| Shadan, 2025 | Physical board game | Moderate | Low | High | Time-intensive design |
| Kavanaugh, 2020 | Escape room (physical + digital) | 7-month planning | High | High | Replication challenges |
| Soylu et al., 2025 | Classroom + QR code video | Moderate | Moderate | Moderate | Single assessor, single site |
| Folz et al., 2025 | CGM device + app + SPs | Moderate (multi-week) | High | Moderate | Device & SP resource needs |
| Twist & Ragsdale, 2022 | Board game (printed) | Low | Low | High | Needs physical space |
| Tan, 2024 | Custom digital game + laptops | High (one-to-one facilitation) | High | Low | High cost; 1:1 ratio limits scalability |

*Abbreviations: NR = not reported; SP = standardized patient; SIM = simulation; CGM = continuous glucose monitor.*
